# Using genotoxic and haematological biomarkers as an evidence of environmental contamination in the Ocoa River native fish, Villavicencio—Meta, Colombia

**DOI:** 10.1186/s40064-016-1753-0

**Published:** 2016-03-22

**Authors:** Wilson Corredor-Santamaría, Marlon Serrano Gómez, Yohana María Velasco-Santamaría

**Affiliations:** Research Group in Aquatic and Environmental Biotechnology and Toxicology - BioTox, Faculty of Agricultural Sciences and Natural Resources, University of the Llanos, Km 12 Vía Puerto López, Villavicencio, Meta Colombia; Colombian Petroleum Institute– ICP, Piedecuesta, Santander Colombia

**Keywords:** *Aequidens metae*, *Astyanax gr. bimaculatus*, Genotoxicity, Haematology

## Abstract

The studies evaluating the impact of environmental pollution on aquatic organisms have had a growing concern in recent decades. The Ocoa River runs through Villavicencio city, receiving the wastewater produced in the city. To date studies reporting the effect and the consequences of Ocoa River pollution on aquatic organisms inhabiting this water body are unknown. Haematological and genotoxicity biomarkers were evaluated in peripheral blood of two native fish species *Astyanax gr. bimaculatus* and *Aequidens metae* during rainy and dry season at three different sites of Ocoa River called Nacimiento (site 1, before entering the city), Centauros (site 2, inside the city), Caño Seco (site 3, after the city). Also, fish from a reference site with little likelihood of contamination were sampled. Alterations such as decrease in erythrocyte count, lymphocytes, haemoglobin concentration and haematocrit percentage, and increased thrombocytes and neutrophils counts, together with nuclear abnormalities in erythrocytes such as increased frequency of micronuclei, lobed, blebbed and notched nuclei and binucleate cells were observed in fish from the site 3, followed by the site 2 in rainy season. These results shown that during rainy season, sites 2 and 3 receive domestic and industrial wastewater leading to genotoxic and haematological changes in the monitored organisms.

**Electronic supplementary material:**

The online version of this article (doi:10.1186/s40064-016-1753-0) contains supplementary material, which is available to authorized users.

## Background

The vast majority of the waste generated by human activities inevitably reaches the water bodies, which, depending on their nature and location, often allow the accumulation of various types of xenobiotics such as polychlorinated biphenyls, organochlorine pesticides, toxic metals, polycyclic aromatic hydrocarbons, dibenzo-p dioxins and polychlorinated dibenzofurans, among others (Van der Oost et al. 2003). Especially in developing countries, the main sources of pollution correspond to domestic and industrial wastewater which are released with little or no treatment to the water bodies. Villavicencio is a city located in the east of Colombia with two main natural water bodies known as Guatiquía River and Ocoa River. The latter receives much of the waste from the city. Unfortunately, no studies revealing the impact of the degradation of this river on aquatic organisms are known. Native fish have been used in numerous studies in situ as bioindicators (Adams and Ham 2011; Mosesso et al. 2012). *Aequidens* and *Astyanax* are freshwater fish species used in biomonitoring of both lentic and lotic waters (Corredor-Santamaría et al. 2012; Schulz and Martins-Junior 2001; Videira et al. 2011).

The use of physiological biomarkers to determine haematological variables or genotoxicity alterations estimating the occurrence of micronuclei in peripheral blood are frequently used as diagnostic techniques to establish the health status of the fish exposed to a complex mixture of available pollutants in water bodies. Studies have been performed showing the relevance of using in situ haematology studies as a tool to reveal the effects of exposure to the discharge of wastewater with high levels of toxic metals such as iron and mercury in the shad *Prochilodus lineatus* (Cazenave et al. 2009), tomoyo *Labrisomus philippii* (Montenegro and González 2012), parrotfish, *Scarus ghobban,* grouper *Epinephelus merra* and rabbit fish *Siganus sutor* (Elahee and Bhagwant 2007). The effect of treated and untreated sewage derived from various industries on Indian carp *Labeo rohita* in lakes have shown a decrease in erythrocyte count, haemoglobin concentration and haematocrit values together with increased white blood cell count as a result of exposure to synthetic detergents, oil and acid and alkaline substances from nearby local industries (Zutshi et al. 2010). Similarly, characterization of damage to genetic material produced by genotoxic compounds has been widely used in conjunction with haematology studies. Seriani et al. (2011) evaluated the effect of the season in San Francisco River in Brazil who found that in summer the frequency of micronuclei and other nuclear abnormalities in Curimatá *Prochilodus argenteus*, painted catfish *Pimelodus maculatus* and Pacú *Myleus micans* increased. These reports demonstrate the relevance of haematological and genotoxic techniques in ecotoxicological studies. Therefore, the aim of this study was to determine the in situ effect of Ocoa River pollution using haematological and genotoxicity biomarkers in *Astyanax gr. bimaculatus* (Pisces: Characidae) and *Aequidens metae* (Pisces: Cichlidae), native fish of the Ocoa River, Villavicencio, Meta, Colombia.

## Methods

### Location and description of study area

**Fig. 1:**
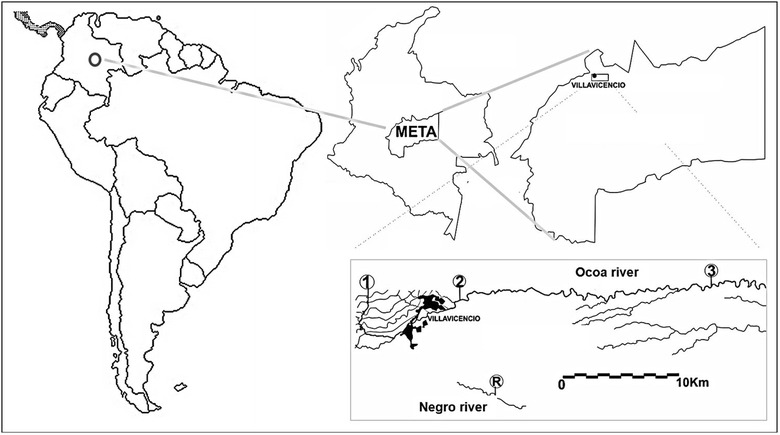
Location map of the monitoring sites on the Ocoa River: *Site 1* (Nacimiento, before entering the city), *site 2* (Centauros, where sewage from the city is dumped), *site 3* (Caño Seco, after the city and close to a landfill) and reference site: (*R*)

Monitoring sites at the Ocoa River, Villavicencio, Meta are shown in the Fig. 1. Fish and water samples were collected at three sites on the river: site 1, called Nacimiento (4°06′09.39″N–73°42′10.70″O) before entering the city; site 2, called Centauros (4°06′15.39″N–73°37′54.71″O) where sewage is dumped; Site 3, called Caño Seco (4°06′53.29″N–73°26′12.13″O) close to a landfill. Similarly, a reference site (R) with little likelihood of contamination, Negro River (4°01′52.55″N–73°36′09.41″O) was monitored.

### Biological material and sampling method

The sampled fish were identified at the Institute of Natural Sciences of the National University of Colombia. The classification corresponded to *Aequidens metae*—ICN 18884 and *Astyanax gr. bimaculatus*—ICN 18885. Twelve fish of each species were caught at each monitoring site. Live fish were collected using conventional fishing net (small square fishing net).

The monitoring was done during dry season (February 2012 and March 2013) and rainy season (October 2012 and May 2013). During each sampling event, water samples were collected to determine the concentration of cadmium and mercury (atomic absorption spectrometry), surfactants (spectrophotometry) and total hydrocarbons (liquid–liquid extraction) in the Water Laboratory at the Industrial University of Santander, Colombia.

Likewise, the physicochemical water characteristics were monitored at each site and each sampling season, including temperature (°C), dissolved oxygen (mg/L), pH, conductivity (mS/cm) and TDS (mg/L) using for this purpose a multiparameter probe (YSI Professional Plus, Ohio USA) and a pH meter (Waterprof Hanna, Mauritius). Alkalinity (mg/L), hardness (mg/L) and ammonium (NH_3_, mg/L) were determined with a HACH kit.

### Blood extraction

Immediately after capture and in order to minimize the stress generated by manipulation, the fish were anesthetized by immersion in a solution of 2-phenoxyethanol (300 ppm, JT Baker, Phillisburg, USA). Afterwards, fish were weighed (digital Ohaus Scout Pro^®^) and the fork length measured. The blood was collected by puncture of the caudal vessels with a 1 ml syringe (using 25Gx5/8″ gauge needle) with heparin as anticoagulant (Handy and Depledge 1999).

The blood was stored in 500 µL Eppendorf tubes and kept on ice until its analysis on the same day of collection. The procedures were done under the laws for the use of laboratory animals described for the Committee on Care and Use of Laboratory Animal Resources—National Research Council, USA (1996) and also allow for the Welfare Committee from the University of the Llanos.

### Haematology

In each sample, a completed haemogram was made. For the blood cells total count (erythrocytes, leukocytes and thrombocytes), a Natt-Herrick solution was used (Conroy 1998) in a 1:200 dilution using a Neubauer chamber (Optic Labor, Germany).

The haematocrit was quantified by centrifugation (7.000 g during 5 min). The haemoglobin concentration was determined by the cyanmethemoglobin method with an absorbance of 540 nm (Jenway 6405 UV/VIS spectrophotometer, Barloworld scientific Ltd. Dunmow, England) using a commercial kit (Spinreact, S.A., Spain). The erythrocytes index such as mean corpuscular volume (MCV), mean haemoglobin corpuscular (MHC) and mean corpuscular haemoglobin concentration (MCHC) were determined following the methodology proposed by Conroy and Conroy (1987).

The differenttial leucocyte count was done in a blood smear stained with Wright-Methanol (Merck^®^) and observed at 100X magnification counting 100 cells per smear (Weiss and Wardrop 2010).

### Genotoxicity

To determine the frequency of micronuclei (MN) and other nuclear abnormalities, two smears were done per fish. These smears were stained with Wright-Methanol (Merck^®^) previously filtered during 10 min. In each smear, 2000 cells with intact nuclear and cytoplasmic membranes were counted (Al-Sabti and Metcalfe 1995). The criteria to determine MN were: MN diameter smaller than 1/3 of the principal nuclei, clearly separated to the nuclei, nonrefractory, with the same colour and intensity and included in the cytoplasm (Grisolia 2002). The nuclear abnormalities were classified according to Carrasco et al. (1990). A blebbed nucleus was identified as a small invagination of the nuclear membrane. Invaginations bigger than the blebbed nuclei with various lobules were classified as lobed nuclei and erythrocytes with a noticeable depression into the nucleus without nuclear material were classified as notched nuclei. Cells with two separated nuclei with similar size were distinguished as binuclear cells. The frequency of micronuclei and other nuclear abnormalities were determined per each fish and expressed in a 1000 cell count (%).

### Lipid peroxidation determination and histopathology

Immediately after blood collection, fish were desensitized by medullary court immediately after blood collection. Liver (n = 6) of each species were sampled and a portion of each was fixed in 10 % buffered formaldehyde for histopathological analysis. The remaining tissue was aliquoted and transported in vapour nitrogen (Dry shipper Taylor-Wharton^®^) and stored at −70 °C until analysis.

Lipid peroxidation (LPO) was determined using the FOX assay (ferrous oxidation xylenol) (Jiang et al. 1991) with minor modifications. Histopathological changes in liver were determined following the severity codes described by Velasco-Santamaría et al. (2011).

### Statistical analysis

A descriptive statistical analysis was done expressing the data as mean ± SEM. Homogeneity of variance (Levene’s or Bartlett test) and data normal distribution (Kolmogorov–Smirnov) were done. A two-way ANOVA was done to evaluate the effect of the different sampling sites and the season on the variables followed by a Tukey test to detect differences between treatments. Transformation was done when data was abnormal and with heterogeneous variance, however, when data did not fulfil those tests, a ranking transformation was done. In all cases, a p value <0.05 was used to show statistical significant differences. The statistical procedures were done in SAS for Windows version 9.02 (2002–2006, SAS Institute Inc., Cary, NC, USA) and in GraphPad v 5.0.

## Results

### Water analysis and physic-chemical parameters

The contaminant concentration evaluated, except for mercury, was within the levels accepted by the Colombian legislation in water bodies (Act 1594/84). Temperature, dissolved oxygen, conductivity and ammonium had significant differences compared to the reference site (p > 0.05, Table 1).

**Table 1:** Physicochemical parameters monitoring in the Ocoa River, Villavicencio—Meta, Colombia

Water physicochemical parameters	Site 1		Site 2		Site 3		Reference	
	Rainy	Dry	Rainy	Dry	Rainy	Dry	Rainy	Dry
Temperature (°C)	22.24*	23.55*	27.57	28.75*	29.33	29.77	30.53	28.7
pH	4.84	5.19	4.41	5.33	5.23	6.52	5.21	5.95
Dissolve oxygen (mg/L)	6.54	7.34	4.42*	3.56*	5.67	3.29*	6.77	5.96
Alkalinity (mg/L)	17.1	17.1	28.5	19.95	65.55	45.6	22.8	22.8
Hardness (mg/L)	62.7	54.15	65.55	51.3	45.6	59.85	22.8	31.35
Total dissolved solids (g/L)	97.83	89.03	86.08	83.1	133.67	133.12	37.05	31.88
Conductivity (µS/cm)	71.54*	121.02*	105.06*	106.05*	86.21*	148.45*	44.4	52.75
Total ammonia (mg/L)	0.11	0.14	0.83*	1.03*	0.61*	1.8*	0.16	0.15
Water chemical analysis								
Cadmium (mg Cd/L)	–	<LD	–	<LD	–	<LD	–	<LD
Mercury (µg Hg/L)	<LD	–	<LD	–	2.37	–	<LD	–
Surfactants (mg SAAM/L)	0.049	<LD	0.053	<LD	0.061	<LD	0.037	<LD
Total hydrocarbons (mg/L)	3.39	4.61	4.53	3.02	<LD	<LD	0.05	<LD

*DL* detectable limit. Site 1 (Nacimiento, before entering the city), site 2 (Centauros, where sewage from the city is dumped), site 3 (Caño Seco, after the city and close to a landfill) and reference site (R)

* For the same season of year, asterisks indicate statistical significant differences (p < 0.05) between the reference site and the monitoring sites. Site 1 (Nacimiento), site 2 (Centauros), site 3 (Caño Seco) and reference site (R)

### Morphometric variables in sampled fish

In the first sampling site was not possible to find *Aequidens metae* species, therefore data for this species in this site are not shown. The lowest weight (1.44 g) and length (4.6 cm) were observed in *A. gr bimaculatus* sampled at the site 3 which corresponds to the farthest site from the city. On the other hand, *A. metae* sampled at the site 2 had the lowest weight (10.83 g) and length (7.34 cm). In both species, the higher weight and length were observed in fish sampled at site 1 for *A. gr. bimaculatus* (3.97 g and 6.17 cm, respectively) and at the reference site for *A. metae* (20.30 g and 9.50 cm, respectively).

### Haematological variables

A significant reduction in the erythrocyte count was observed during the rainy season in *A. metae* sampled at site 2 and site 3 (p < 0.05) when compared to the reference site (Table 2). On the other hand, a significant reduction in the haemoglobin concentration was observed in dry season in *A. metae* sampled at sites 2 and 3 (p < 0.05) when compared to the reference site (Table 2). Likewise, in *A. gr bimaculatus* a significant decrease in haemoglobin concentration was observed in fish sampled during dry season at site 2 site compared to site 1 and reference site (p < 0.05, Table 3).

**Table 2:** Haematological parameters of *Aequidens metae* caught in different sites of the Ocoa River and a reference site (Negro river), Villavicencio—Meta, Colombia during dry and rainy season

Nuclear alteration	Dry			Rainy		
	Site 2	Site 3	Reference	Site 2	Site 3	Reference
Erythrocytes (x106x µl-1)	2.06 ± 0.09	2.09 ± 0.10	2.38 ± 0.13	1.58 ± 0.09*	1.80 ± 0.12*	2.26 ± 0.15
Leukocytes (x103x µl-1)	71.65 ± 6.71	74.25 ± 5.95	60.11 ± 5.46	40.09 ± 1.35	56.56 ± 1.85	32.32 ± 1.62
Thrombocytes (x103x µl-1)	70.10 ± 9.04	51.24 ± 4.67	57.36 ± 6.96	33.59 ± 1.33	52.75 ± 2.23*	26.73 ± 0.88
Hemoglobin (g/dl)	7.82 ± 0.31*	7.47 ± 0.32*	10.03 ± 0.27	8.55 ± 0.18	8.15 ± 0.08	8.61 ± 0.51
PVC (%)	21.0 ± 0.68	19.26 ± 0.79*	23.63 ± 0.94	23.57 ± 0.85	21.60 ± 0.46*	26.0 ± 0.98
MCV (fL)	107.38 ± 5.33	105.90 ± 8.40	105.04 ± 8.21	153.80 ± 4.01	138.20 ± 7.0	119.73 ± 5.79
MCH (pg)	41.03 ± 2.75	41.68 ± 3.53	44.33 ± 2.93	59.18 ± 3.60*	55.09 ± 3.85*	35.56 ± 5.46
MCHC (%)	37.70 ± 1.42	40.14 ± 1.78	43.0 ± 1.34	37.91 ± 1.78*	38.63 ± 1.10*	28.30 ± 2.97
Lymphocytes (%)	63.27 ± 0.50*	56.62 ± 0.50*	75.38 ± 1.05	63.18 ± 0.53*	64.93 ± 1.57*	73.93 ± 1.08
Neutrophils (%)	29.30 ± 0.84*	32.41 ± 0.74*	23.75 ± 0.90	28.75 ± 1.05*	28.98 ± 0.96*	23.0 ± 0.95
Monocytes (%)	3.03 ± 0.28	2.51 ± 0.16	2.38 ± 0.29	3.46 ± 0.31	2.98 ± 0.16	2.79 ± 0.32
Basophils (%)	0.55 ± 0.09	0.59 ± 0.08	0.44 ± 0.13	0.57 ± 0.10	0.40 ± 0.08	0.64 ± 0.13
Eosinophils (%)	0 ± 0	0 ± 0	0.38 ± 0.13	0 ± 0	0.30 ± 0.07	0.36 ± 0.13

* For the same season of year, asterisks indicate statistically significant differences (p < 0.05) between the reference site and the monitoring sites. Site 1 (Nacimiento, before entering the city), site 2 (Centauros, where sewage from the city is dumped), site 3 (Caño Seco, after the city and close to a landfill) and reference site (R)

**Table 3:** Haematological parameters of *Astyanax gr. bimaculatus* caught in different sites of the Ocoa River and a reference site (Negro river), Villavicencio—Meta, Colombia during dry and rainy season

Nuclear alteration	Dry				Rainy			
	Site 1	Site 2	Site 3	Reference	Site 1	Site 2	Site 3	Reference
Erythrocytes (x106x µl-1)	1.83 ± 0.14	1.65 ± 0.10	1.73 ± 0.08	2.18 ± 0.21	1.56 ± 0.05	1.33 ± 0.07	1.43 ± 0.08	1.55 ± 0.11
Leukocytes (x103x µl-1)	62.26 ± 4.56	56.83 ± 7.15	64.14 ± 8.28	57.48 ± 4.25	32.46 ± 1.80	35.83 ± 1.53	39.70 ± 1.74	39.53 ± 1.20
Thrombocytes (x103x µl-1)	62.82 ± 6.96	53.45 ± 9.63	48.37 ± 9.98	50.93 ± 7.43	28.65 ± 2.21	30.53 ± 1.80	35.44 ± 2.06	32.41 ± 1.15
Hemoglobin (g/dl)	10.87 ± 0.38	7.91 ± 0.33*	8.65 ± 0.58	10.08 ± 0.36	10.79 ± 0.22	9.78 ± 0.28	9.41 ± 0.21	10.36 ± 0.20
PVC (%)	24.27 ± 0.73	23.91 ± 0.78	24.43 ± 0.66	25.30 ± 0.93	22.66 ± 0.87	21.35 ± 0.80	23.48 ± 0.91	22.94 ± 1.14
MCV (fL)	144.34 ± 6.82	152.59 ± 6.25	145.53 ± 5.11	133.50 ± 9.35	145.71 ± 3.11	163.97 ± 4.36	167.63 ± 3.85	152.43 ± 5.69
MCH (pg)	70.45 ± 6.34	54.62 ± 5.19	54.31 ± 5.58	56.78 ± 5.24	71.93 ± 2.99	78.78 ± 5.41	68.79 ± 2.61	71.83 ± 4.68
MCHC (%)	46.75 ± 2.73	34.51 ± 2.32	36.67 ± 3.04	41.57 ± 2.59	49.97 ± 2.34	47.64 ± 2.74	41.09 ± 1.36	47.09 ± 2.54
Lymphocytes (%)	70.81 ± 0.49	57.45 ± 1.04*	54.05 ± 1.14*	70.09 ± 0.68	70.25 ± 0.58	55.65 ± 1.19*	52.56 ± 0.84*	69.76 ± 0.85
Neutrophils (%)	20.23 ± 0.66	24.18 ± 0.78*	28.90 ± 1.04*	20.00 ± 0.57	19.38 ± 0.60	25.00 ± 0.83*	27.40 ± 0.76*	19.47 ± 0.82
Monocytes (%)	5.65 ± 0.49	3.91 ± 0.45	4.33 ± 0.40	5.61 ± 0.49	5.78 ± 0.46	4.10 ± 0.36	4.00 ± 0.29	6.35 ± 0.62
Basophils (%)	0.42 ± 0.10	0.36 ± 0.10	0.48 ± 0.11	0.48 ± 0.11	0.53 ± 0.09	0.55 ± 0.11	0.48 ± 0.10	0.47 ± 0.12
Eosinophils (%)	0.46 ± 0.10	0.36 ± 0.10	0.38 ± 0.11	0.35 ± 0.10	0.44 ± 0.09	0.35 ± 0.11	0.60 ± 0.10	0.35 ± 0.12

* For the same season of year, asterisks indicate statistically significant differences (p < 0.05) between the reference site and the monitoring sites. Site 1 (Nacimiento, before entering the city), site 2 (Centauros, where sewage from the city is dumped), site 3 (Caño Seco, after the city and close to a landfill) and reference site (R)

In both seasons, the PVC was significantly higher in the reference site compared to the other sites in *A. metae* fish (Table 2). Contrarily, this trend was not observed in *A. gr bimaculatus*. In *A. metae* sampled during rainy season was observed a significant increase in the MCH and MCHC percentage at the two sampling sites compared to the reference site (Table 2).

The thrombocyte count in *A. metae* sampled during rainy season at the site 3 had a higher count with statistically significant differences (p < 0.05) compared to the reference site (Table 2). In both seasons, a significant decrease was observed in the lymphocytes count from *A. metae* sampled at sites 2 and 3 compared to the reference site (p < 0.05, Table 2). Similarly, in *A. gr. bimaculatus* sampled in both seasons, a significant decrease in the lymphocytes count was observed in fish sampled at sites 2 and 3 compared to the site 1 and reference sites (p < 0.05, Table 3). On the other hand, the neutrophils count had a different trend in both species during both seasons, where a significant increase was observed in fish sampled at sites 2 and 3 compared to the Nacimiento and reference sites (p < 0.05, Tables 2, 3).

### Genotoxicity

**Fig. 2:**
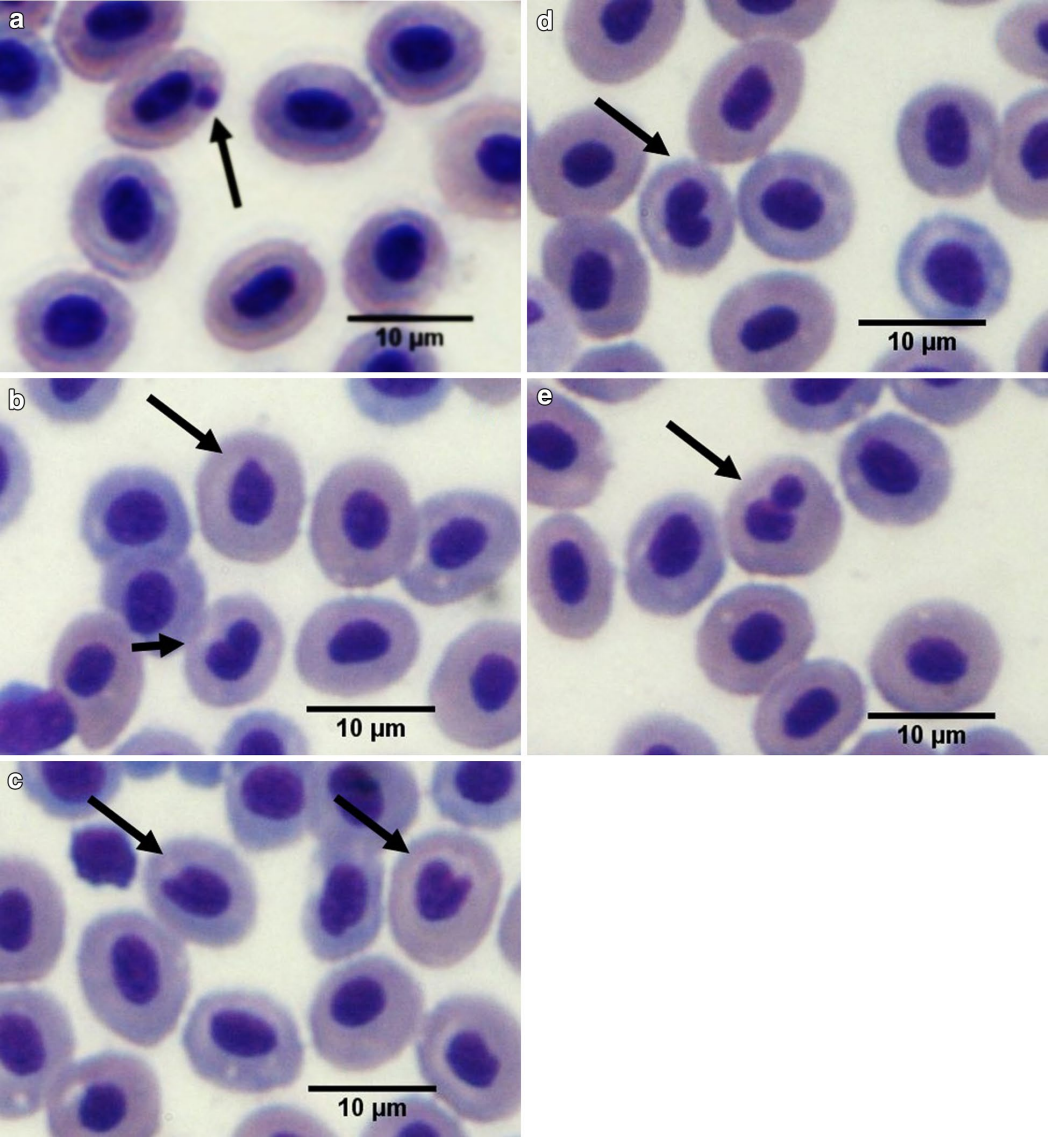
Erythrocytes with micronuclei (**a**) lobed nuclei (**b**), blebbed nuclei (**c**), notched nuclei (**d**) and binucleated cell (**e**) in peripheral blood *Astyanax gr. bimaculatus* and *Aequidens metae*. *Scale bar* 10 µm. Wright stain

In *A. gr bimaculatus* and *A. metae* peripheral blood, erythrocyte with elliptical nuclei in a central position with defined contour were classified as normal erythrocytes. A genotoxic effect characterized by higher frequency of micronuclei, lobed, blebbed and notched nuclei and binucleated cells (Fig. 2), was observed during both seasons and in both species caught at site 2 and site 3 with significant differences when compared to reference and Nacimiento sites (Table 4).

**Table 4:** Frequency of micronuclei, lobed, blebbed and notched nuclei and binucleated cells in peripheral blood of *Astyanax gr. bimaculatus* and *Aequidens metae* sampled in different sites of Ocoa River and a reference site (Negro River), Villavicencio—Meta, Colombia during dry and rainy season

Specie	Nuclear alteration	Dry				Rainy			
		Site 1	Site 2	Site 3	Reference	Site 1	Site 2	Site 3	Reference
*Astyanax gr. bimaculatus*	MN	0.07 ± 0.01	0.28 ± 0.03*	0.35 ± 0.04*	0.12 ± 0.01	0.09 ± 0.01	0.27 ± 0.03*	0.36 ± 0.03*	0.09 ± 0.02
	Lobed	0.09 ± 0.02	0.37 ± 0.04*	0.44 ± 0.04*	0.09 ± 0.02	0.09 ± 0.01	0.42 ± 0.4*	0.51 ± 0.04*	0.11 ± 0.02
	Blebbed	0.11 ± 0.02	0.40 ± 0.03*	0.48 ± 0.04*	0.05 ± 0.01	0.08 ± 0.01	0.36 ± 0.04*	0.41 ± 0.04*	0.10 ± 0.01
	Notched	0.10 ± 0.02	0.66 ± 0.04*	0.71 ± 0.05*	0.07 ± 0.01	0.08 ± 0.01	0.73 ± 0.03*	0.74 ± 0.03*	0.11 ± 0.02
	Binucleated cell	0.08 ± 0.01	0.44 ± 0.04*	0.46 ± 0.05*	0.08 ± 0.01	0.07 ± 0.01	0.47 ± 0.04*	0.50 ± 0.05*	0.12 ± 0.01
*Aequidens metae*	MN	**–**	0.33 ± 0.02*	0.39 ± 0.01*	0.06 ± 0.01	**–**	0.36 ± 0.03*	0.37 ± 0.03*	0.09 ± 0.02
	Lobed	**–**	0.32 ± 0.02*	0.42 ± 0.02*	0.08 ± 0.01	**–**	0.41 ± 0.03*	0.44 ± 0.02*	0.09 ± 0.01
	Blebbed	**–**	0.34 ± 0.03*	0.41 ± 0.03*	0.07 ± 0.01	**–**	0.35 ± 0.03*	0.40 ± 0.01*	0.11 ± 0.02
	Notched	**–**	0.59 ± 0.03*	0.71 ± 0.03*	0.09 ± 0.02	**–**	0.74 ± 0.02*	0.70 ± 0.04*	0.08 ± 0.02
	Binucleated cell	**–**	0.38 ± 0.02*	0.40 ± 0.03*	0.12 ± 0.02	**–**	0.47 ± 0.04*	0.49 ± 0.03*	0.15 ± 0.03

* For the same season of year, asterisks indicate statistically significant differences (p < 0.05) between the reference site and the monitoring sites. Site 1 (before entering the city), site 2 (where sewage from the city is dumped), site 3 (after the city and close to a landfill)

### Lipid peroxidation determination and histopathology

Liver lipid peroxidation was significantly higher at the site 1 compared to the control group in rainy season (Additional file 1: Figure S1). The main liver histopathological changes are illustrated in Additional file 1: Figure S2. Hepatic vacuolization was more intense in sites 2 and 3. Similarly, inflammatory (congestion), lamellar and interlaminar hyperplasia, and epithelial lifting aneurysms were observed in sites 2 and 3 in both fish species with more alterations during the dry season. A detailed description of liver and gill histology alterations will be shown in another manuscript.

## Discussion

This study is a pioneer in the region since in situ determination of the effects of water pollution on native fish species inhabiting the Ocoa River has not previously evaluated. It is important to highlight that although the water data analysis are below the limits allowed by the Colombian environmental legislation, these minimum concentrations exceed those values accepted in other countries such as USA, which use research reports constantly carry out in their own natural water bodies as a criterion for the selection of the allowed limits in the dumping of industrial sewage and domestic wastewater (EPA 2009).

It is possible that the lowest temperature monitored at the site 1 (Nacimiento) of the Ocoa River did not allow adaptation of the *A. metae* species, therefore, no fish from this species was found at this site.

The reduction in erythrocyte count in *A. metae* at Centauros (site 2) and Caño Seco (site 3) during the rainy season could be explained to the possible decrease in the haematopoiesis caused by intrasplenic and intrahepatic haemorrhage generated for the water contaminants such as heavy metals (Zaghloul et al. 2007). These results are in agreement with those observed in this study, in where higher hepatic congestion in both seasons was observed at site 2 and site 3 (Additional file 1: Figure S2).

Likewise, the reduction in erythrocyte count and haematocrit percentage in *A. metae* at Caño Seco (site 2) and Centauros (site 3) in rainy season and the decrease in haemoglobin concentration in both sites in dry season could be due to the presence of high levels of ammonia and toxic metals such as mercury (Ishikawa et al. 2007) or copper (Kumar and Nandan 2014), being these contaminants able to produce haemolysis in fish (Zaghloul et al. 2007). An study from Çavaş (2008) showed that mercury not only interferes with the proliferation of erythrocytes but also inhibit the release of polychromatic and normochromatic erythrocytes to the peripheral circulation. An haemolytic effect of common contaminants in water bodies receiving wastewater containing for example pyrethroid insecticides has been associated with inhibition of the Na^+^K^+^-ATPase activity, leading to an increase in the sodium influx into the cells producing disturbances in the ion exchange affecting cell permeability which induce cell swelling and finally cell membranes rupture (Assis et al. 2009). On the other hand, the decrease in haemoglobin concentration observed in contaminated sites along the Ocoa River could be due to an increase in the haemoglobin destruction or reduction in the rate of haemoglobin synthesis due to the presence of toxic metals such as cadmium, mercury and lead (Senthamilselvan et al. 2012).

The increase in the MCH and MCHC concentration observed in *A. metae* during rainy season at Centauros (site 2) and Caño Seco (site 3) when compared to the reference site can be associated with feedback responses of membranes erythrocytes structural damage as a result of the hemolysis and impaired haemoglobin synthesis and also to the erythrocytes release stress due to the hypoxia observed at sites 2 and 3. Similar results were reported by Kayode and Shamusideen (2010) who exposed Nile tilapia (*Oreochromis niloticus*) to sublethal concentrations of diesel (23.4 mg/L) and drilling fluid (492 mg/L) for 28 days.

The reduction in the erythrocyte count, haemoglobin concentration and haematocrit percentage found in this study are associated with the inflammatory (congestion) and cellular alterations (lamellar and interlamellar hyperplasia, aneurysms and epithelial lifting) observed in this study (Additional file 1: Figure S2). Those results probably are due to the hypoxia induced by contaminants that interfere with the gas exchange capacity. These results are supported by the ones observed by Moharram et al. (2011) in *Siganus rivulatus* exposed to different concentrations of sea water nearby a sewage from the Egyptian Mediterranean coast and also to the study done by Elahee and Bhagwant (2007) in *Scarus ghobban* exposed to polluted waters of the lagoon Bain des Dames, Mauritius.

The haematological disorders were associated to the disturbances in liver morphology evidenced as presence of pyknotic nuclei, vacuolization and lymphocytic infiltration, similar to the results found by Maceda-Veiga et al. (2010) in *Barbus meridionalis* caught in different areas of Ripoll River, which receives high volumes of wastewater, being those alterations higher when higher concentrations of toxic metals and poor water quality conditions were observed.

Thrombocytes are associated with the response of cellular defence in teleost fish (Tavares-Dias et al. 2007). In this study the increase in both thrombocytes and leukocyte count in *A. gr. metae* fish sampled at site 3 during rainy season and the reduction of those parameters in dry season could be due to the availability of pollutants stimulating the increase in the defence response during the rainy season (Seriani et al. 2011), while in dry season lead to inhibition (Jerônimo et al. 2009; Tavares-Dias et al. 2008).

Lymphocytes are considered the most important cells of the immune response since contain in their membrane receptors able to recognise certain antigenic molecules (Ruíz et al. 2003) and correspond to the higher percentage of leukocytes. The lymphopenia observed in *A. gr. bimaculatus* and *A. metae* in both seasons could be due to the stress induced by the water pollution. Ishikawa et al. (2007) observed that the stress in Nile tilapia leads to lymphocytes redistribution, reduction in the lymphocytes blood circulation and also a probable lymphocytes destruction in response to the high cortisol levels. On the other hand, neutrophils are phagocytic cells and its increase (neutrophilia) could be due to a higher phagocytic activity in the presence of pollutants (Weiss and Wardrop 2010). In addition, Oliveira Ribeiro et al. (2006) reported that in *Hoplias malabaricus* exposed to MeHg, the neutrophilia could be the result of tissue damage. In this study, lymphopenia and neutrophilia were observed which could be attributed to the exposure to different pollutants such as heavy metals (Montenegro and González 2012), sewage (Maceda-Veiga et al. 2010) and industrial effluents (Akinrotimi et al. 2013), among others.

Several studies have found a direct relationship between the occurrence of lipid peroxidation (LPO) and DNA damage expressed as MN presence (Ali et al. 2014; Nwani et al. 2010). In the present study, the occurrence of lipid peroxidation and liver histopathological alterations were observed in *A. gr. bimaculatus* from Caño Seco (site 3) and Centauros (site 2) during both seasons, being these findings together with higher genotoxic damage evidenced by the presence of micronuclei, lobed nuclei, notched nuclei and binuclear erythrocytes. The relation between LPO and genotoxicity could be attributed to the fact that in the presence of damage in the lipid cell membrane, the genetic material is expose and can be trigger irreversible mutations due to a hydrogen deletion in the DNA, being this reaction higher than the capacity to restore the DNA. Supporting this finding, a study from Katsumiti et al. (2013) evaluated the impact of the oil spill in a water body 5 years after the accident and found similar results than the observed in the present study. Additionally, rainy season has been associated to higher micronuclei presence (Ossana and Salibián 2013), similar to the observed effect in the present study, evidencing that during the rainy season there is a greater availability of genotoxic compounds.

It has been suggested that problems in segregating tangled and attached chromosomes or gene amplification via the Breakage–Fusion–Bridge cycle could cause lobed nuclei or blebbed nuclei during the elimination of amplified DNA from the nucleus (Çavaş 2008). Likewise, the occurrence of lobed and notched nuclei has been attributed to the presence of substances able to induce cytotoxicity and it is recommended to quantify thus abnormalities separately (Bolognesi and Hayashi 2011). At this regard, the higher presence of lobed and notched nuclei in both fish species sampled at the site 3 indicates the presence of cytotoxic damage in peripheral erythrocytes; furthermore, based on our results it is possible to infer that *A. gr. bimaculatus* is more sensitive to the formation of this type of nuclear abnormalities.

Finally, several studies have shown that the occurrence of binuclear erythrocytes is associated to the presence of wastewater from thermal power plants in *Labeo bata* and *Oreochromis mossambica* (Talapatra et al. 2007), and also in *Oreochromis niloticus*, *Oreochromis aureus* and *Tilapia zilli* exposed in situ to domestic and industrial sewage discharged to the Nile (Ali et al. 2008); therefore the induction of this abnormality has been considered as an indicator of cytotoxicity (Çavaş and Ergene-Gözükara 2015). In the present study, the sites of the Ocoa River closest to the city and receiving domestic wastewater without treatment lead to the highest incidence of binucleate erythrocytes, indicating a greater exposure of the biota to cytotoxic pollutants in different concentrations leading to genotoxic effects (García-Medina et al. 2011).

In conclusion, the results obtained demonstrated haematological and genotoxic effects in fish caught at site 2 and site 3, areas in which wastewater from the city of Villavicencio are discharged. This effect could have a negative impact in the decline of fish populations inhabiting the Ocoa River. Finally, the information obtained through this study using bioindicators and sensitive biomarkers can be useful for the Colombian Government agencies to legislate on the sewage dumping.

## Additional file

10.1186/s40064-016-1753-0 Lipid peroxidation occurrence in liver of *Astyanax gr. bimaculatus* captured in different sites of the Ocoa River and a reference site (Negro River) during dry and rainy season. Site 1 (Nacimiento, before entering the city), site 2 (Centauros, where sewage from the city is dumped) and site 3 (Caño Seco, after the city and close to a landfill). ^a,b,c^ Bars with different letters indicate significant statistical differences (p<0.05) between monitoring sites for the same season. **Figure S2.** Histopathological changes in liver alterations of *Aequidens metae* with core pyknotic (>) and vacuolization (°). Scale bar 50 µm. H & E stain.
