# Supplementary material for: Using genotoxic and haematological biomarkers as an evidence of environmental contamination in the Ocoa River native fish, Villavicencio—Meta, Colombia
Source: Springerplus. 2016 Mar 22;5:351. doi: 10.1186/s40064-016-1753-0 (PMC4801821; doi:10.1186/s40064-016-1753-0)

ADDITIONAL FILE

Fig. S1 Lipid peroxidation occurrence in liver of *Astyanax gr. bimaculatus* caught in different sites of the Ocoa River and a reference site (Negro River) during dry and rainy season. Site 1 (Nacimiento, before entering the city), site 2 (Centauros, where sewage from the city is dumped) and site 3 (Caño Seco, after the city and close to a landfill).

[a,b,c] Bars with different letters indicate significant statistical differences ($p<0.05$) between monitoring sites for the same season.

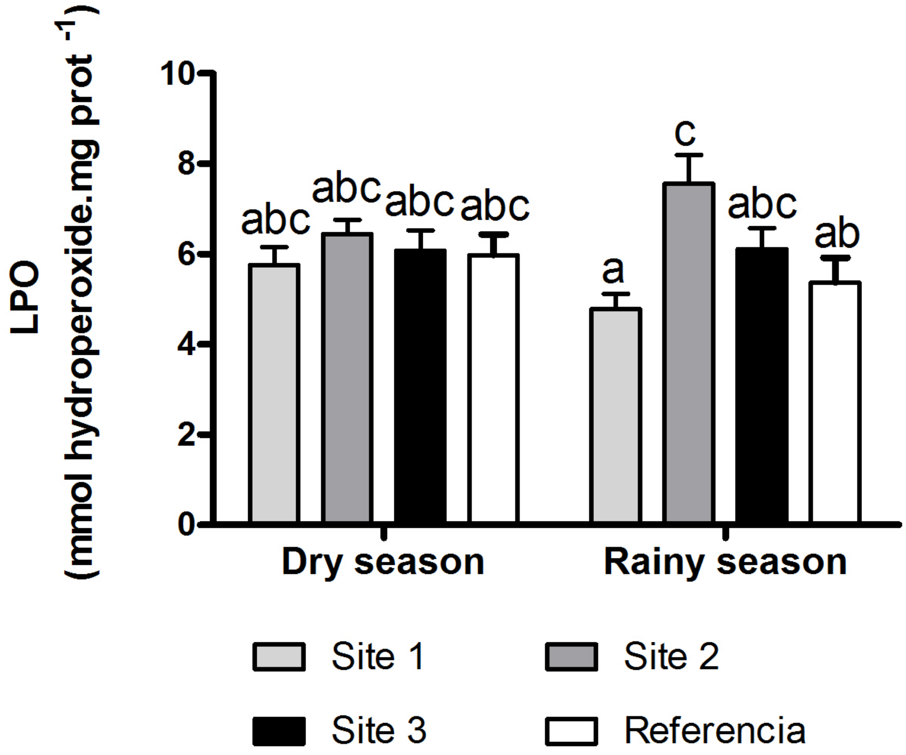

Fig. S2 Histopathological changes in liver of *Aequidens metae* showing pyknotic core (>) and vacuolization (°). Scale bar 50 μm. H & E stain.

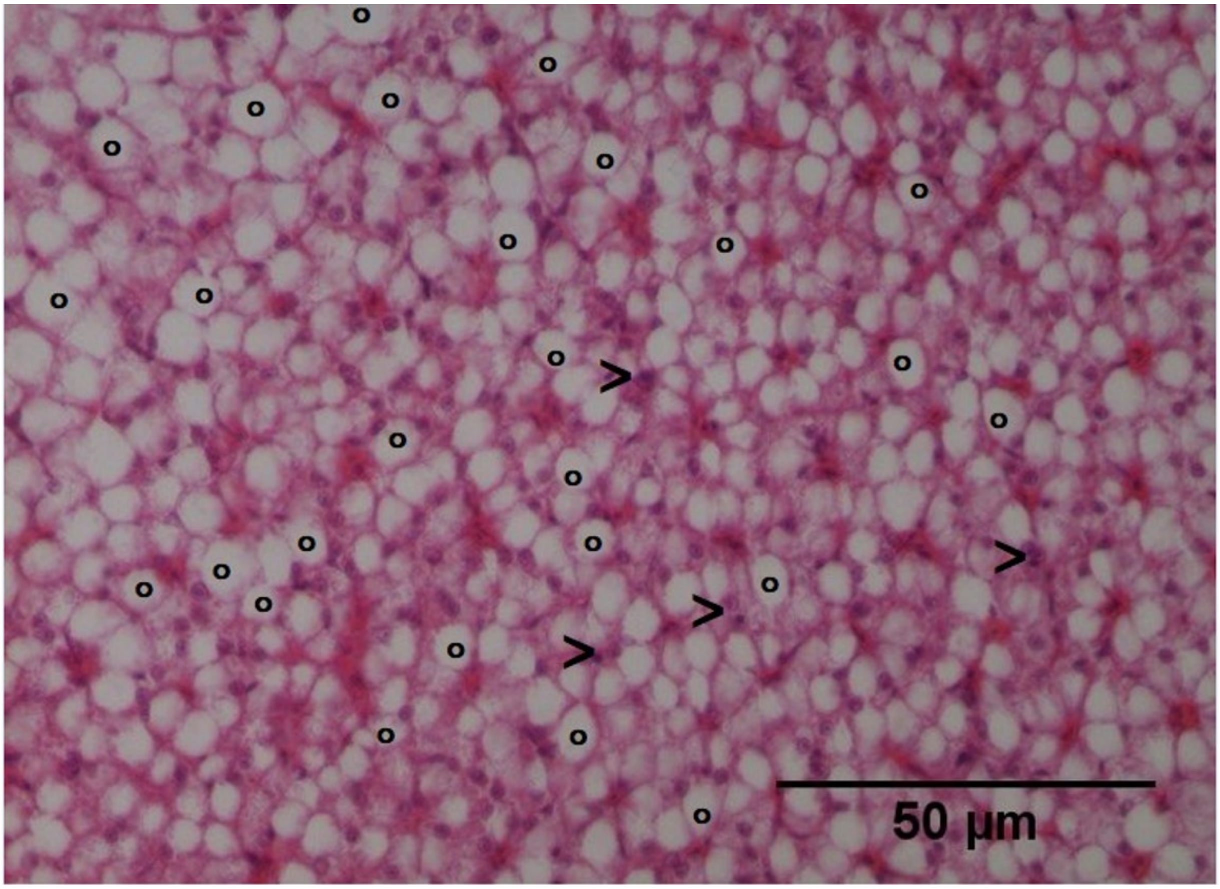

Supplement: Supplementary file 1 — 10.1186/s40064-016-1753-0 Lipid peroxidation occurrence in liver of Astyanax gr. bimaculatus captured in different sites of the Ocoa River and a reference site (Negro River) during dry and rainy season. Site 1 (Nacimiento, before entering the city), site 2 (Centauros, where sewage from the city is dumped) and site 3 (Caño Seco, after the city and close to a landfill). a,b,c Bars with different letters indicate significant statistical differences (p<0.05) between monitoring sites for the same season. Figure S2. Histopathological changes in liver alterations of Aequidens metae with core pyknotic (>) and vacuolization (°). Scale bar 50 µm. H & E stain. [file 40064_2016_1753_MOESM1_ESM.pdf]
